# Studies on Autophagy and Apoptosis of Fibrosarcoma HT-1080 Cells Mediated by Chalcone with Indole Moiety

**DOI:** 10.3390/ijms25116100

**Published:** 2024-06-01

**Authors:** Ewelina Honkisz-Orzechowska, Olga Barczyk-Woźnicka, Maria Kaleta, Jadwiga Handzlik, Katarzyna Kieć-Kononowicz

**Affiliations:** 1 Faculty of Pharmacy, Department of Technology and Biotechnology of Drugs, Jagiellonian University Medical College, 9 Medyczna Street, 30-688 Kraków, Poland; ewelina.honkisz@uj.edu.pl (E.H.-O.); maria.kaleta@uj.edu.pl (M.K.); j.handzlik@uj.edu.pl (J.H.); 2 Laboratory of Transmission Electron Microscopy, Department of Cell Biology and Imaging, Institute of Zoology and Biomedical Research, Jagiellonian University, 9 Gronostajowa Street, 30-387 Kraków, Poland; olga.woznicka@uj.edu.pl

**Keywords:** fibrosarcoma, autophagy, LC3-II, apoptosis, indole-based chalcone, MIPP, MOMIPP, mitochondrial membrane potential

## Abstract

This study demonstrated the anticancer efficacy of chalcones with indole moiety (MIPP, MOMIPP) in fibrosarcoma cells for the first time. The results showed that MIPP and MOMIPP reduced the viability of HT-1080 cells in a concentration-dependent manner. MOMIPP was more active than MIPP in HT-1080 cells, showing lower IC_50_ values (3.67 vs. 29.90 μM). Both compounds at a concentration of 1 μM induced apoptosis in HT-1080 cells, causing death strictly related to caspase activation, as cell viability was restored when the caspase inhibitor Z-VAD was added. Reactive oxygen species production was approximately 3-fold higher than in control cells, and cotreatment with the inhibitor of mitochondrial ATPase oligomycin diminished this effect. Such effects were also reflected in mitochondrial dysfunction, including decreased membrane potential. Interestingly, the compounds that were studied caused massive vacuolization in HT-1080 cells. Immunocytochemical staining and TEM analysis showed that HT-1080 cells exhibited increased expression of the LC3-II protein and the presence of autophagosomes with a double membrane, respectively. Both compounds induced apoptosis, highlighting a promising link between autophagy and apoptosis. This connection could be a new target for therapeutic strategies to overcome chemoresistance, which is a significant cause of treatment failure and tumour recurrence in fibrosarcoma following traditional chemotherapy.

## 1. Introduction

Sarcomas are a heterogeneous group of mesenchymal origin divided into two main types: approximately 80% arise from soft tissue, and 20% originate from bone [1]. Soft tissue sarcomas are a challenge in treatment due to their significant genotypic and phenotypic heterogeneity. Indeed, there are about 100 different subtypes according to genetics, pathology, anatomical location, and clinical behaviour, including gastrointestinal stromal tumours, rhabdomyosarcoma, osteosarcoma, Ewing’s sarcoma, leiomyosarcoma, liposarcoma, synovial sarcoma, and fibrosarcoma [2]. Fibrosarcoma is a tumour of mesenchymal cell origin that can occur as a rare form of soft tissue cancer [3]. The World Health Organization defined fibrosarcoma as a malignant neoplasm composed of fibroblasts with variable collagen production and, in classical cases, a ‘herringbone architecture’ [4]. The combination of surgical treatment with radiation therapy and sometimes chemotherapy still represents the backbone of systemic treatment. In general, patients are prescribed chemotherapy drugs (i.e., doxorubicin, ifosamide, cisplatin, carboplatin, and etoposide) in the early stages and radiation therapy and surgeries in the later and severe stages. The results of a meta-analysis involving 14 clinical trials and 1568 patients with sarcoma undergoing postoperative chemotherapy with doxorubicin showed an improvement in relapse-free survival by 10% (significant difference) and overall survival by approximately 6% (no significant difference) [5]. In the past decade, very few new drugs have been approved that offer some survival benefits. Tazemetostat (Food and Drug Administration, FDA approved in Jan 2020), a specific histone-lysine N-methyltransferase enzyme inhibitor (enhancer of zeste homolog 2, EZH2) is the first targeted treatment for epithelioid sarcoma with a median progression-free survival of ~6 months and a median overall survival of ~1.5 years [6]. A new treatment approach was taken with nab-sirolimus, a nanoparticle of an mTOR inhibitor that received FDA approval in November 2021. This treatment showed considerably greater tumour accumulation, suppression of the mTOR target, and antitumour activity [7]. Treatment with nab-sirolimus resulted in a median progression-free survival ~11 months and median overall survival ~3.5 years. New immunological strategies for the treatment of sarcomas have been also developed under the IMMUNOSARC phase II clinical trial, in which the synergistic efficacy of the anti-PD-1 (nivolumab) plus the antiangiogenic agent (sunitinib) was explored [8]. Anthracyclines and alkylating agents are still the main medications used to treat fibrosarcoma. However, using radiation therapy and chemotherapy to treat fibrosarcoma is not always sufficient. Therefore, it is essential to find new targets for this tumour entity.

Researchers at the University of Toledo discovered a series of synthetic chalcones (indolyl-pyridinyl-propenones; IPP) capable of inducing a non-apoptotic form of cell death called methuosis [9,10]. The characteristic features of methuosis are massive cellular vacuolization and the loss of cellular metabolic integrity [11]. Two the most active compounds, MIPP (3-(2-Methyl-1-H-indol-3-yl)-1-(4-pyridinyl)-2-propen-1-one) and MOMIPP (3-(5-methoxy-2-methyl-1H-indol-3-yl)-1-(4-pyridinyl)-2-propen-1-one), were able to induce methuosis and reduce cell viability in temozolomide-resistant U251 human glioblastoma cells. Most vacuoles were shown to originate from clathrin-independent macropinosomes because the presence of filipin (endocytosis inhibitor) blocks vacuole induction.

**Figure 1:**
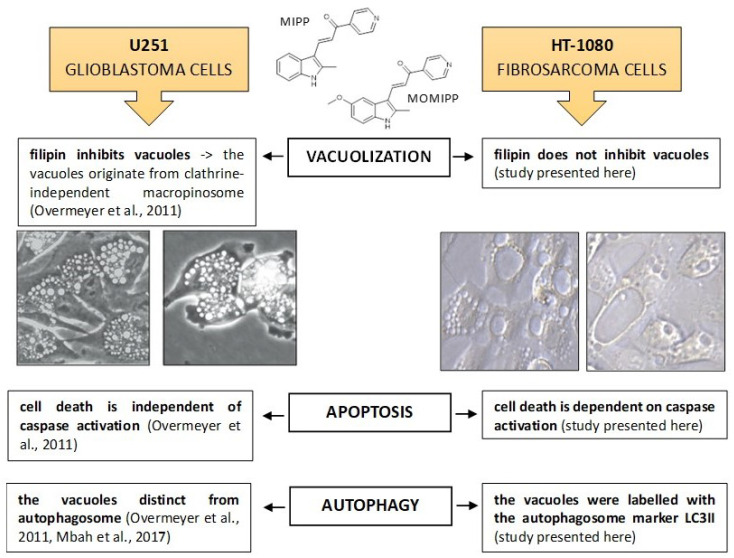
Different actions of MIPP and MOMIPP in glioblastoma cells and fibrosarcoma cells [9,12]. Scale bar of microphotographs is 10 microns.

In our study, we found that the compounds described earlier also caused the formation of large vacuoles in HT-1080 fibrosarcoma cells, leading to cell death. However, the characteristics of these vacuoles were slightly different. The vacuole membranes exhibited markers of the autophagosome, specifically LC3-II. Furthermore, we observed that HT-1080 cells treated with MIPP or MOMIPP displayed the significant activation of caspases. Interestingly, treatment with a pan-caspase inhibitor (Z-VAD-fmk) resulted in the complete restoration of cell viability. A summary of the differences in the mechanisms of the actions of MIPP and MOMIPP compounds in glioblastoma and fibrosarcoma cells is shown in Figure 1. These observations led us to conduct more detailed research on the role of cytoplasmic vacuolization in cell death.

## 2. Results

### 2.1. Indole-Based Chalcones Induced Massive Vacuolization and Loss of Cell Viability

**Figure 2:**
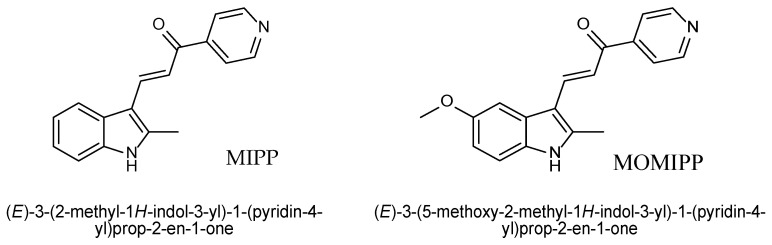
The structure of MIPP and MOMIPP.

**Figure 3:**
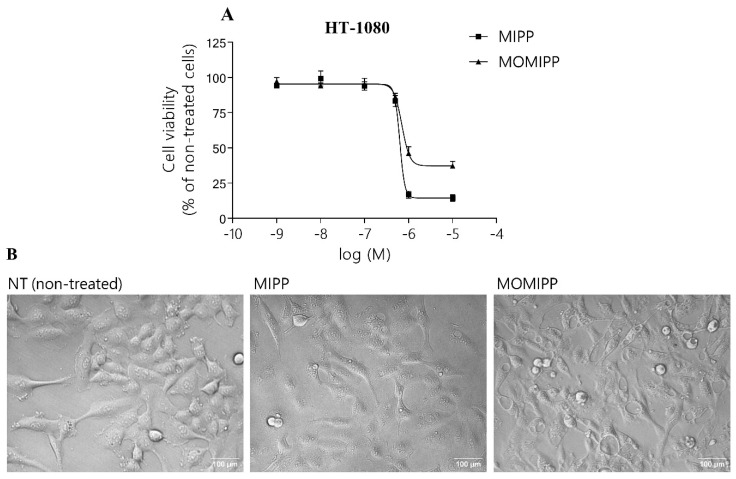

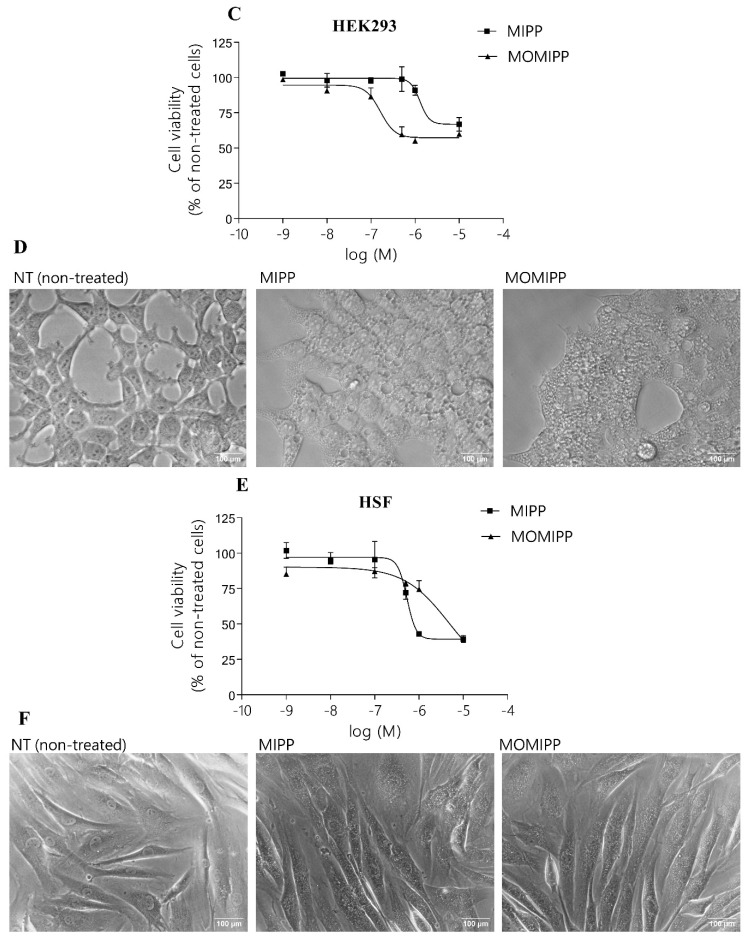
Effect of MIPP and MOMIPP on cell viability and morphology. HT-1080, HEK293, and HSF cells were incubated for 24 h in the presence of increasing concentrations of MIPP or MOMIPP (1 × 10^−9^–25 × 10^−6^). (**A**,**C**,**E**) Cell viability was measured by MTS assay. Each point represents the mean ± SEM of three independent experiments, comprising six replicates per treatment group. (**B**,**D**,**F**) Cells were photographed with a phase-contrast microscope.

The cell viability was measured over a wide range of concentrations (from 1 nM to 25 μM of MIPP and MOMIPP (Figure 2, [9]). Our results demonstrated that the cell viability was reduced in HT-1080, HEK293, and HSF cells by MIPP and MOMIPP in a concentration-dependent manner (Figure 3A,C,E). The half-maximum inhibitory concentration (IC_50_) values for MIPP and MOMIPP are presented in Table 1. The results showed that MOMIPP was more active than MIPP in HT-1080 cells that showed lower IC_50_ values (3.67 vs. 29.90 μM), while MIPP was much more active than MOMIPP in immortalized HEK293 cells and normal human skin fibroblast HSF (1.29 and 0.56 vs. 16.28 and 9.70 μM, respectively). Our results are similar to those obtained by Robinson et al. [10]. MOMIPP was more effective than MIPP in reducing the cell growth and viability of HSF and glioblastoma cancer cells. However, in our studies, MOMIPP at a concentration of 10 μM still slightly precipitated in the culture medium. Morphologically, exposure to MIPP and MOMIPP resulted in identical massive cellular vacuolization in all cells tested (Figure 3B,D,F). Although the vacuoles in HT-1080 looked different, they were larger than those in HSF. In contrast to Overmeyer et al., the vacuoles in our tested cell lines did not originate from macropinosomes, as filipin (endocytosis inhibitor) did not block their formation [9]. We also observed that the cells cultured in the presence of MIPP or MOMIPP were still adherent even after 72 h. Interestingly, removing MIPP and MOMIPP after 24 h of treatment did not prevent vacuole formation, indicating a non-reversible effect of the treatment of these two compounds.

### 2.2. MIPP and MOMIPP-Induced Cell Death Is Dependent on Caspase Activation

**Figure 4:**
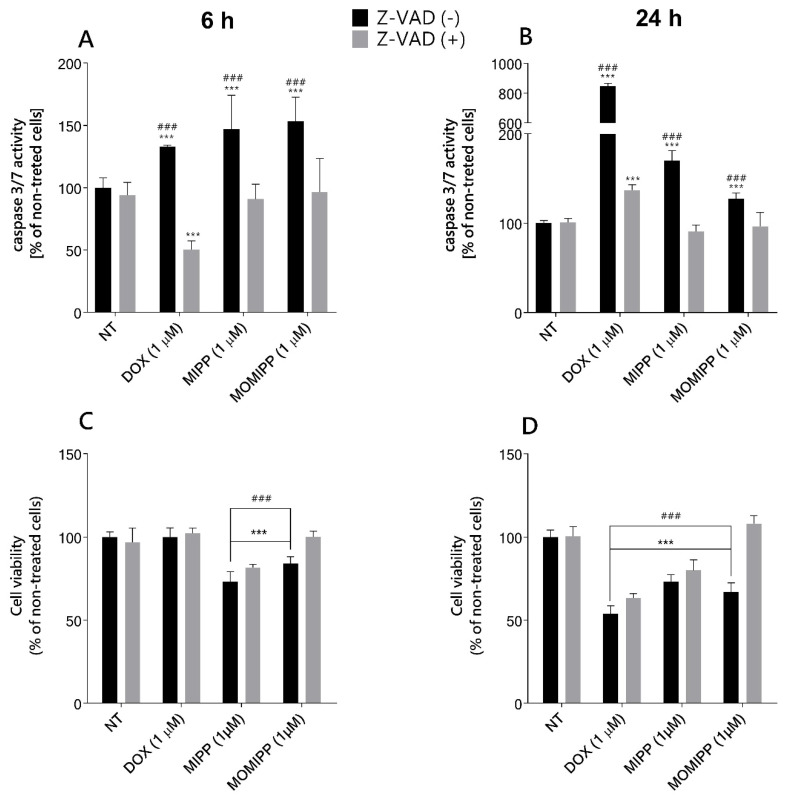
Effect of MIPP and MOMIPP on caspase-3/7 activity and cell viability in HT-1080 cells. HT-1080 cells were incubated for 6 h (**A**,**C**) and 24 h (**B**,**D**) in the presence of MIPP or MOMIPP (1 μM) with or without Z-VAD(OMe)-FMK (10 μM). The caspase-3/7 activity was determined by Apo-ONE^®^ Homogeneous Caspase-3/7 Assay, and cell viability was determined by MTS assay. Each point (mean ± SEM of two independent experiments, each of which consisted of eight replicates per treatment group) represents the relative fluorescence units (RFU) and is expressed as a percentage of NT (nontreated cells) set as 100%. The statistical analysis by one-way ANOVA showed significant differences between the groups (α = 0.05) and was followed by Sidak’s multiple comparison test. Data indicated with *** *p* ≤ 0.001 (versus NT) and ^###^ *p* ≤ 0.001 (versus Z-VAD treated cells) reflect statistically significant differences.

As Overmeyer et al. reported, U251 glioblastoma cells treated with MIPP showed some caspase activation, which was unnecessary for cell death [9]. However, in light of our observations, the HT-1080 cells possessed some characteristic features of apoptosis, namely, membrane blebbing. We reexamined whether the mechanism of action of MIPP and MOMIPP is distinct from apoptosis. The activity of caspase-3/7 was assessed to determine the type of cell toxicity of MIPP and MOMIPP in HT-1080 cells. Our data revealed the activation caspase-3/-7 in cells treated with 1 μM of MIPP or MOMIPP as compared to the vehicle control (Figure 4A,B). MIPP and MOMIPP at doses of 1 μM caused significant activation of caspase-3/7, comparable after 6 h and 24 h treatment. The percentage of caspase-3/7 activation showed a 1.5-fold increase in MOMIPP- and MIPP-treated cells. We added a broad-spectrum caspase inhibitor (Z-VAD(OMe)-FMK) to establish whether cell death solely relied on caspase activation. Our results demonstrated that the caspase inhibitor Z-VAD(OMe)-FMK blocked the initiation of apoptosis by MIPP or MOMIPP, confirming that cell death relied on caspase activity. Caspase-3 is known for its role in the execution of apoptosis, but no apparent properties are specific to caspase-3 [13]. Caspase-3 plays many roles in cell biology, and its levels can affect cell death and survival. Even though Z-VAD(OMe)-FMK can inhibit caspase-3 and possibly restore its levels in the cell, this may not necessarily mean that the viability of the cell will return. Using the same experimental condition, we examined cell viability. Interestingly, the cells treated with MIPP and caspase inhibitor did not completely restore cell viability, although the level of caspase-3 was the same as in the nontreated cells (Figure 4C,D). The opposite effect was observed in the MOMIPP-treated cells, where decreased caspase-3 activity increased cell viability. This might indicate a distinct mechanism of cell death: Z-VAD (OMe)-FMK-sensitive.

### 2.3. Exposure to MIPP or MOMIPP Triggers Reactive Oxygen Species (ROS) Production

**Figure 5:**
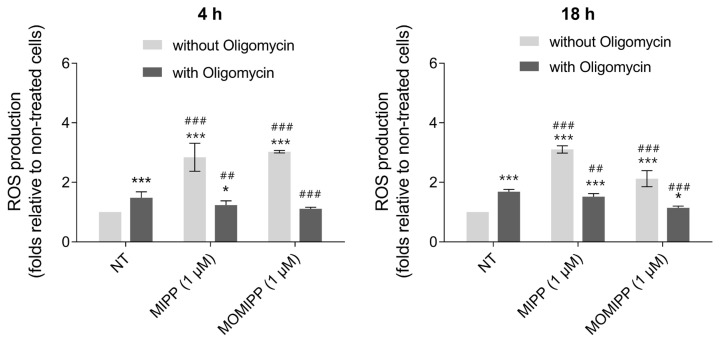
Effect of MIPP and MOMIPP on ROS production in HT-1080 cells. HT-1080 cells were incubated for 4 h and 18 h in the presence of MIPP or MOMIPP (1 μM) with or without oligomycin (30 µM). Each point (mean ± SEM of three independent experiments, each of which consisted of six replicates per treatment group) represents the relative fluorescence units (RFU) and is expressed as a fold change of NT (nontreated cells). The statistical analysis by one-way ANOVA showed significant differences between the groups (α = 0.05) and was followed by Sidak’s multiple comparison test. Data indicated with * *p* ≤ 0.033, *** *p* ≤ 0.001 (versus NT without Oligomycin) and ^##^ *p* ≤ 0.002, ^###^ *p* ≤ 0.001 (versus NT with Oligomycin) reflect statistically significant differences.

HT-1080 cells treated with 1 µM of MIPP or MOMIPP alone showed increased ROS production that was significantly higher than in the nontreated cells and remained at a similar level after short- and long-term incubation (Figure 5). It is known that mitochondrial respiration is the primary source of free radicals [14]. Mitochondria are also the site for ATP generation in oxidative phosphorylation. That is why the inhibitor of ATP synthase, oligomycin, was used to study the process of ROS induction. Oligomycin alone also induced ROS production in a short- or long-term incubation but to a lesser extent than MIPP or MOMIPP alone. A cotreatment with oligomycin reduced ROS production following the treatment with MIPP or MOMIPP. These results regarding the restoration of ROS levels otherwise elevated by MIPP and MOMIPP showed the specific site of ROS production. Adding oligomycin to MIPP- or MOMIPP-treated cells dramatically decreased the level of ROS to that for oligomycin alone. 

### 2.4. Changes in the Mitochondrial Membrane Potential Play a Role in Fibrosarcoma Cell Death Triggered by MIPP and MOMIPP

**Figure 6:**
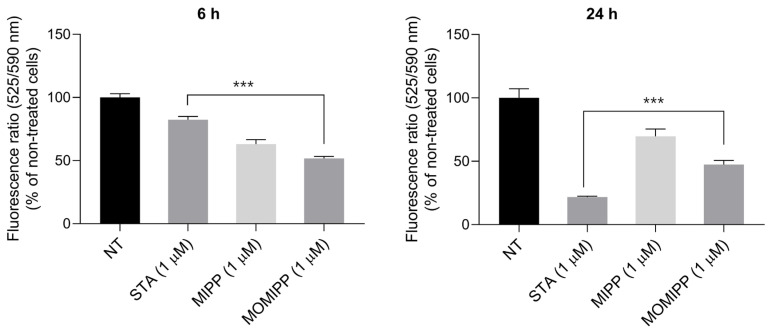
Effect of MIPP and MOMIPP on mitochondrial membrane potential in HT-1080 cells. HT-1080 cells were incubated for 6 h and 24 h in the presence of MIPP or MOMIPP (1 μM). Staurosporine (STA) served as a positive control in this assay. Each point (mean ± SEM of three independent experiments, each of which consisted of four replicates per treatment group) represents the relative fluorescence units (RFU) and is expressed as a fold change of NT (nontreated cells). The statistical analysis by one-way ANOVA showed significant differences between the groups (α = 0.05) and was followed by Dunnett’s multiple comparison test. Data indicated with *** *p* ≤ 0.001 reflect statistically significant differences between NT and experimental groups.

Mitochondrial pathways play a significant role in vertebrate apoptosis [15]. As seen in Figure 6, MIPP and MOMIPP at the concentration of 1 μM were able to depolarise the mitochondrial membrane, which might contribute to apoptosis induction. The results showed an almost 50% decrease in potential as compared to the nontreated cells, which remained at a similar level after 6 h and after 24 h. This study used staurosporine (STA) at a concentration of 1 μM, a well-known apoptosis inducer, as a positive control.

### 2.5. MOMIPP-Induced Vacuoles Acquire Characteristics of Autophagosomes

**Figure 7:**
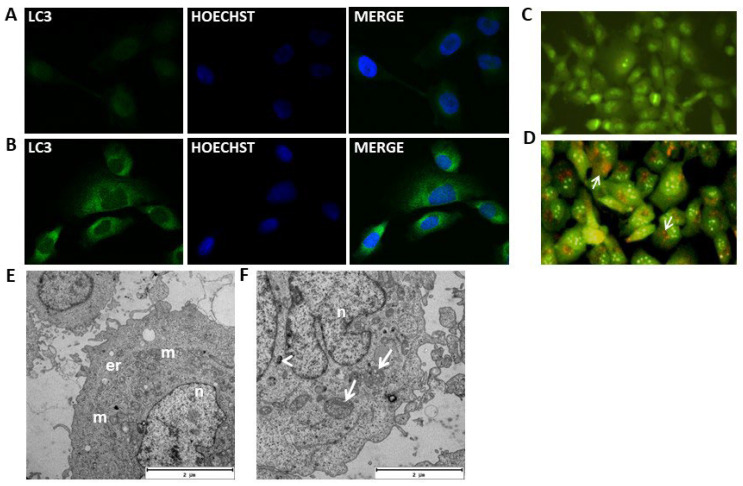
Effect of rapamycin on HT-1080 cells. ((**A**,**B**), magnification 200×) Representative confocal fluorescence microphotographs for LC3-II detection. Cells were treated for 24 h with 0.1% of DMSO (vehicle) (**A**) or with 500 nM of rapamycin (**B**). The green punctuated fluorescence indicates the cytoplasmic localization of the LC3-II proteins (1:200 NL006 R&D Systems, 2 h of incubation, λ_EX_ = 493 nm, λ_EM_ = 514 nm). The representative micrographs demonstrated characteristic punctuate staining indicative of autophagosome formation (left panel). The blue fluorescence indicates the nuclear location and was shown by Hoechst counterstaining (1 μg/mL, 10 min incubation, λ_EX_ = 352 nm, λ_EM_ = 454 nm, middle panel). The images merged from LC3-II and Hoechst are shown in the right panel. (**C**,**D**) Representative fluorescence images of Acridine Orange (AO, λ_EX_ = 490 nm, λ_EM_ = 520 nm)-stained HT-1080 control cells (**C**, magnification 200×), treated with 500 nM of rapamycin (**D**, magnification 200×) for 24 h. Microphotograph (**D**) indicates the formation of Acridine Orange-accumulating autophagic vacuoles (orange-red fluorescence, white arrows). Green and red fluorescence were detected in the AO-stained cells using a fluorescence microscope. (**E**,**F**) Representative Transmission Electron Microscopy (TEM) images of HT-1080 cells treated with DMSO (vehicle) (**E**) or with 500 nM of rapamycin (**F**). The control HT-1080 cells (**E**) have intact cellular morphology with ellipsoidal nuclei (n) containing non-condense chromatin. The mitochondria (m) showed normal ultrastructures with typical tubular cristae, and the granular endoplasmic reticulum (er) is well developed. The rapamycin-treated cells (**F**) displayed autophagosomes with a double membrane (white arrowhead) and autophagolysosomes with a single membrane (white arrows).

**Figure 8:**
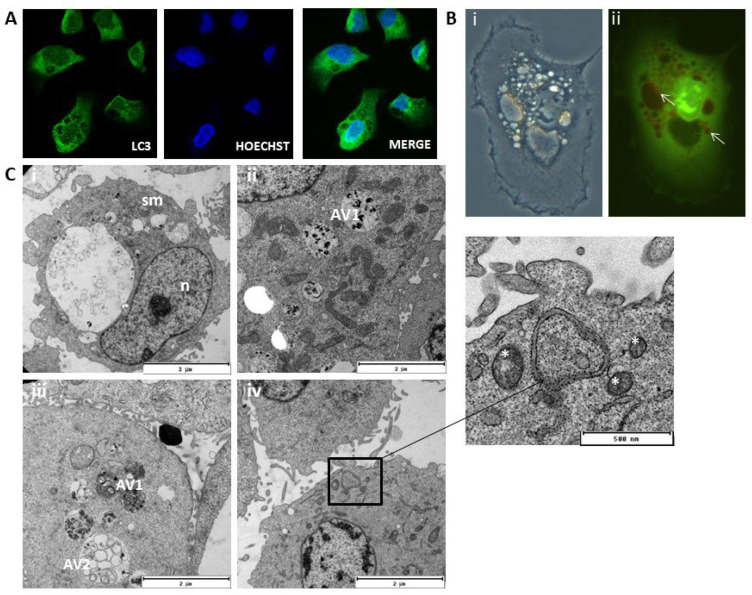
Effect of MOMIPP on HT-1080 cells. ((**A**), magnification 200×) Representative confocal fluorescence microphotographs for LC3-II detection. Cells were treated for 24 h with 1 µM of MOMIPP. The green punctuated fluorescence indicates the cytoplasmic localization of the LC3-II proteins (1:200 NL006 R&D Systems, 2 h of incubation, λ_EX_ = 493 nm, λ_EM_ = 514 nm). The representative micrographs demonstrated characteristic punctuate staining indicative of autophagosome formation (left panel). The blue fluorescence indicates the nuclear location (n) and was shown by Hoechst counterstaining (1 μg/mL, 10 min incubation, λ_EX_ = 352 nm, λ_EM_ = 454 nm, middle panel). The images merged from LC3-II and Hoechst are shown in the right panel. ((**B**), magnification 200×) Phase-contrast (**i**) and fluorescence (**ii**) microscopy of HT-1080 cells treated for 24 h with 1 µM of MOMIPP. MOMIPP treatment causes numerous large vacuoles (**i**,**ii**, white arrows). (**C**) TEM images of HT-1080 cells treated with 1 µM of MOMIPP. Swollen mitochondria (sm) and abnormal morphology were observed (**i**). Several large autophagic vacuoles at various stages of development dominate the cytoplasm (**ii**,**iii**). Some are filled with membranous whorls (AV1), while others contain scattered vesicles of various sizes (AV2). Autolysosomes with double-layered membranes contained mitochondria (white asterisk) (**iv**).

MOMIPP (IC_50_ = 3.67 μM), as well as MIPP (IC_50_ = 29.90 μM), induced numerous vacuoles in the cytoplasm of HT-1080 cells, but for a deeper analysis of the nature of these vacuoles, the more active compound MOMIPP was chosen. As can be seen in Figure 7C, untreated (control) cells displayed primarily green fluorescence when stained with acridine orange. The cells treated with MOMIPP have vacuoles of different sizes. Some of them (especially the large ones) showed similarity to macropinosomes. In contrast, the others were stained with acridine orange and formed acidic vesicular organelles (AVOs) characterized by red fluorescence (Figure 8B(ii)). Based on this preliminary experiment, it can be presumed that MOMIPP induced the forming of autophagic vacuoles. However, this acidotrophic dye can also be retained in other intracellular acidic compartments [16]. To confirm the hypothesis that the studied compound induces autophagy in HT-1080 cells, we performed immunocytochemical staining for the presence of the microtubule-associated protein light chain 3 (LC3), which is a widely used marker for autophagosomes [17]. In this case, LC3 is expressed as a propeptide, which is cleaved to form LC3-I. During autophagy activation, LC3-I is diffused in the cytoplasm and converted to LC3-II, localized on the autophagosomal membranes. Thus, the detection of LC3-II can be used to estimate the abundance of autophagosomes [18]. First, rapamycin was applied to induce autophagy in the HT-1080 cells as a positive control. The confocal microscopic analysis demonstrated that, after rapamycin treatment for 24 h, the green punctuated fluorescence formed indicates the cytoplasmic localization of the LC3-II proteins (Figure 7B) compared to the control cells treated with DMSO 0.1% (Figure 7A). These results are consistent with previous reports that the morphological changes in autophagic vacuole formation and the autophagy ratio analyzed by flow cytometry were markedly increased after treatment with rapamycin [19]. Electron microscopic analysis (TEM) also confirmed the presence of autophagosomes with a double membrane and autophagolysosomes with a single membrane (Figure 7F). The effects of MOMIPP on LC3-II protein expression were then examined in parallel (Figure 8A). Immunodetection confirmed the results of acridine orange staining (Figure 8B(ii)), and the vacuoles were labelled with the autophagosome marker—LC3-II protein (Figure 8A). The characteristic punctuated fluorescence was most numerous on the smaller vacuole’s outside membrane, while the larger vacuole’s interior remained empty. TEM analysis revealed that after MOMIPP treatment for 24 h, several large autophagic vacuoles at various stages of development dominate the cytoplasm of the HT-1080 cells (Figure 8C). While the control cells exhibited nuclei with finely dispersed chromatin surrounded by cytoplasm with normal-appearing mitochondria (Figure 7E), abnormalities in the cell morphology were observed after MOMIPP treatment including swollen mitochondria, some of which were surrounded by double-layered membranes (Figure 8C(i–iv)). This would indicate some features of mitophagy.

### 2.6. The Autophagy Inhibitor Chloroquine Increases the Sensitivity of HT-1080 Cells to MIPP and MOMIPP

**Figure 9:**
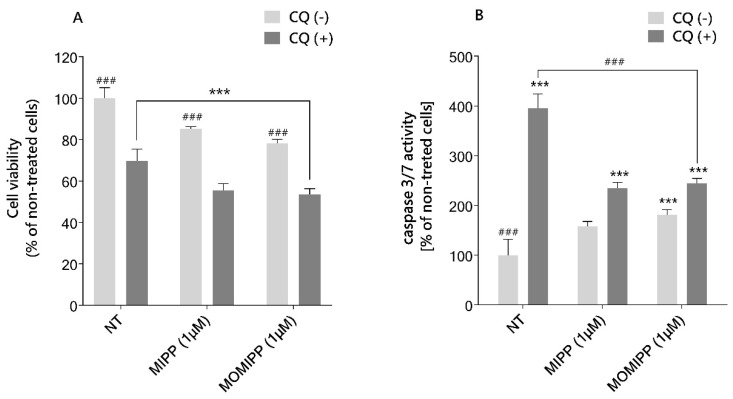
Autophagy inhibitor chloroquine increases the sensitivity of HT-1080 cells to MIPP and MOMIPP. The HT-1080 cells were incubated for 15 h in the presence of MIPP or MOMIPP (1 μM), with or without chloroquine 60 μM (CQ). Cell viability is presented in panel (**A**), and the activity of caspase 3/7 is presented in panel (**B**). Each point represents the mean ± SEM of three independent experiments, each consisting of four replicates per treatment group. The statistical analysis by one-way ANOVA showed significant differences between the groups (α = 0.05) and was followed by Sidak’s multiple comparison test. Data indicated with *** *p* ≤ 0.001 (versus NT) and ^###^ *p* ≤ 0.001 (versus CQ-treated cells) reflect statistically significant differences.

The previous experiment showed that treating HT-1080 cells with MIPP or MOMIPP induced autophagosome formation. We hypothesized that the blockade of late-phase autophagy might make cells more sensitive to tested compounds. Therefore, cells were cotreated with MIPP or MOMIPP and chloroquine (CQ), which prevents autophagosomal degradation [20]. The MTS assay was then used to test cell viability. The results showed that the inhibition of autophagy flux sensitizes HT-1080 cells to MIPP- and MOMIPP-induced cell death (Figure 9A). On average, cell viability was significantly lower in the presence of CQ by 30% and 20% for MIPP or MOMIPP, respectively, compared to MIPP or MOMIPP alone. It was also found that CQ induced apoptosis in osteosarcoma [21]. The fact that osteosarcoma and fibrosarcoma arise from the same mesenchymal tissue suggests a potential for similar cellular processes. We applied an assay for caspase 3/7 activity to examine whether viability testing is reflected in the activation of apoptosis. As illustrated in Figure 9B, the induction of caspase 3/7 activity was more pronounced in cells co-treated with CQ than in cells treated with MIPP or MOMIPP alone. Nevertheless, apoptosis induction was 1.6-fold lower than for CQ alone.

## 3. Discussion

In this present study, we demonstrated that synthetic indole-based chalcones (MIPP, MOMIPP) reduced the viability of fibrosarcoma HT-1080 cells and induced cell death that was strictly dependent on the caspase activation only in the case of MOMIPP. The most remarkable event immediately after the treatment was the non-reversible effect of massive vacuolization in all cells. Transmission electron microscopy showed that tested compounds induced the formation of autophagic vacuoles, which was further confirmed by LC3-II puncta staining using confocal microscopy. Interestingly, the inhibition of autophagy-flux by chloroquine-sensitized cells to death upon MIPP and MOMIPP treatment was visible in the decreased metabolic activity of the cells and the increased apoptosis level. These are the first studies of this type to show the antitumour effects of indole-based chalcones on fibrosarcoma cells. Previous research reported that specific indole-based chalcones can cause cell death in glioblastoma, gastric carcinoma cells, and triple-negative breast cancer cells MDA-MB-231 [22,23].

The current study found that MOMIPP showed more significant activity than MIPP in fibrosarcoma HT-1080 cells in vitro with lower IC_50_ values (3.67 vs. 29.90 μM). Unfortunately, this compound also showed cytotoxic effects against HSF and HEK293 cells, although the IC_50_ values were higher than those of HT-1080 cells (9.70 and 16.28 μM, respectively). These results confirmed previous research by Robinson et al., in which, after exposure to MOMIPP for 48 h, normal fibroblast viability decreased by 40% in viability [10]. Interestingly, the same study noted that cell viability decreased when fibroblasts were plated at a subconfluent density to maintain exponential growth during exposure. High initial density and, therefore, a stationary phase at the time of exposure resulted in the maintenance of high cell viability. As part of our research, we attempted to examine apoptosis to understand better the reasons behind the decreased metabolic activity of the HT-1080 cells. This kind of research seemed justified because Overmeyer et al. reported that U251 glioblastoma cells treated with MIPP showed some evidence of caspase activation. However, it was unnecessary for cell death [9]. This outcome is contrary to our results, which showed that z-VAD-(OMe)-FMK, a broad-spectrum caspase inhibitor, prevented the loss of viability of MOMIPP-treated HT-1080 cells, unlike MIPP, which caused cell death even when caspase-3 was blocked. In line with previous studies, treatment with MIPP or MOMIPP resulted in massive non-reversible vacuolization, which filled much of the cytoplasmic space. Ultrastructural analyses by transmission electron microscopy (TEM) showed the formation of double or multiple membrane-bound vacuolar structures containing partially digested cytoplasm in HT-1080 cells, demonstrating that MOMIPP induced autophagosome formation. The appearance of autophagy was further characterized by fluorescence confocal microscopy analysis that revealed that the vacuoles were labelled with the autophagosome marker LC3-II. MIPP produced a similar effect, but the intensity was much lower, which was also confirmed with weak diffuse staining with LC3-II. This current study’s findings align with those of a research team from the University of Toledo. They proved that MIPP triggered the formation of vacuoles in glioblastoma cells that expressed markers of late endosomes, such as Rab7 and LAMP1, but did not appear to be lysosomes or autophagosomes. The same group also noticed that glioblastoma cells treated with MOMIPP revealed an increase in punctate LC3-II fluorescence, indicating an increase in the number of autophagosomes [9].

The autophagy process is divided into five distinct stages: initiation, nucleation, expansion and elongation, closure and fusion, and cargo degradation, and each stage has potential clinical targets [24]. The inhibition of autophagy by decreasing autophagosome/lysosome fusion is an excellent example in which current clinically available drugs are used, including chloroquine (CQ) and hydroxychloroquine (HCQ) [20]. As autophagy has been discovered to be a cell survival mechanism in tumour cells, autophagy inhibition began to be seen as a therapeutic target. Moreover, the synergy between autophagy inhibitors and other drugs (chemotherapeutics) could be beneficial. Mice with MDAMB231 xenografts (triple-negative breast cancer cells) treated with CQ and doxorubicin had better event-free survival than the matched controls [25]. Another study also indicated that the inhibition of autophagic flux via CQ improves the effectiveness of osimertinib in MDAMB231 cancer cells in vitro [26]. In turn, proteasome inhibitors combined with the inhibition of autophagy induced more human prostate cancer cell death [27]. The inhibition of autophagy by TPEN (that also chelates zinc) positively affected fibrosarcoma by killing cancer cells selectively [28]. Similar trends were obtained in our study when HT-1080 cells were cotreated with the compound and CQ, and the decline in cell viability was more significant than in the presence of the compound alone. In addition, HT-1080 cells were more susceptible to apoptosis upon treatment with CQ and MIPP or MOMIPP simultaneously. However, the mechanism underlying the antitumour effects of cotreatment with chloroquine is only beginning to be understood. Chloroquine possesses weak base properties, can accumulate in lysosomes, and may trigger apoptosis by inhibiting autophagic protein degradation.

The development of resistance to conventional chemotherapy is a major cause of failure of treatment and tumour recurrence in fibrosarcoma. Therefore, the results presented here are only a prelude to further research to evaluate the importance of crosstalk between autophagy and apoptosis, which is likely a novel therapeutic target for reversing chemoresistance. Our results show that, for the first time, MIPP and MOMIPP can induce caspase-dependent apoptosis in HT-1080 cells. However, while blocking this process resulted in cell death for the MIPP-treated cells, the viability of the MOMIPP-treated cells was not affected when a caspase inhibitor was used. Both compounds induced a marked decrease in mitochondrial membrane potential and an increase in ROS. Interestingly, the level of ROS induction with MIPP or MOMIPP was markedly lower after oligomycin pretreatment, indicating that oxidative phosphorylation is functionally essential in fibrosarcoma HT-1080 cells. These findings align with the statement that mitochondria are the energy source for various biological processes in cancer cells and that ROS results from oxidative phosphorylation [29]. Regarding mitochondrial functions, they are a promising target for developing anticancer drugs. Moreover, it was reported that targeted therapies on oxidative phosphorylation sensitize other therapies, such as radiation or immunotherapies [30]. Second, a more active compound, MOMIPP, induced autophagosome formation and, in turn, the inhibition of autophagy flux significantly sensitized HT-1080 cells to death induced by this compound, which was accompanied by the induction of caspase-3/7 activity and reduced metabolic activity. Interestingly, the TEM analysis also showed some hallmarks of mitophagy. We speculate that this might be due to disturbed autophagic flux with tested compounds, leading to the excessive accumulation of damaged mitochondria. Typically, autophagy helps to keep cellular homeostasis by engulfing damaged organelles like mitochondria, which can be a source of ROS that causes DNA damage. In our study, the death of fibrosarcoma HT-1080 cells was caused by the increased level of ROS, the depolarization of the mitochondrial inner membrane, and the increased number of autophagosomes. These findings align with studies in which the autophagosome accumulation induced mitochondria-dependent apoptosis via the ROS-MAPK pathway in lung cancer cells [31]. It supports the hypothesis that blocked autophagy flux can result in tumour cell death. After all, this paper is not intended to be an exhaustive study of the mechanism of these cell death types and does not explain how inhibiting one of them could impact the other. However, it briefly overviews the conceptual crosstalk between autophagy and apoptosis. Future investigations are needed to verify the conclusions that can be derived from this study.

## 4. Materials and Methods

### 4.1. Cell Culture and Treatment Conditions

Human fibrosarcoma cell line HT-1080 (ATCC^®^ no. CCL-121™), human embryonic kidney cell line HEK293 (ATCC^®^ no. CRL-1573™), and normal human skin fibroblast HSF (kindly provided by Prof. J. Drukała, Department of Cell Biology, Jagiellonian University) were grown in Dulbecco’s modified Eagle’s medium (DMEM, no. 41965039, Gibco^TM^, Thermo Scientific, Paisley, Scotland, UK) with 10% (*v*/*v*) fetal bovine serum (South American origin FBS, no. 10500064, Gibco^TM^, Thermo Scientific, Grand Island, USA) at 37 °C in a humidified atmosphere of 5% CO_2_/95% air. The cells were routinely subcultured at 70% confluence by trypsinization (0.05% Trypsin-EDTA, no. 15400054, Gibco^TM^, Thermo Scientific, Waltham, MA, USA) and reseeded at 5 × 10^3^ cells/cm^2^. We evaluated cells from passages 5–12 to ensure comparability between experiments. For the experiments, the cells were seeded in 100 μL (96-well plates, no. 167008, Nunc^TM^, Thermo Scientific; no. 165305, Nunc^TM^, Thermo Scientific, Denmark) or 25 μL (384-well plates, no. 33396, SPL Life Sciences, Pocheon, South Korea) of complete culture medium and cultured overnight. Then, the medium was aspirated, and fresh medium with tested compounds at the desired concentrations were added. After a sufficient time, cell-based assays were performed as described in the following paragraphs. Indole-based chalcone, MIPP, and MOMIPP (Figure 2, [9]) were synthesized in the Department of Technology and Biotechnology of Drugs according to the literature method [10]. The full purity analysis (TLC, HPLC) and confirmation of structures with spectral methods (UPLC-MS, ^1^H NMR and ^13^C NMR) are described in Supplementary Materials. All tested compounds were dissolved in dimethylsulfoxide (DMSO) to make 10 mM stock and stored in aliquots at −20 °C. Each aliquot was used only once to prevent freeze-thaw effects. Immediately before each experiment, an intermediate solution of the tested compounds was made in DMSO as a 1000-fold stock solution and then diluted in a culture medium to the final desired concentration. The cells were treated with increasing concentrations of MIPP or MOMIPP (1 nM, 10 nM, 100 nM, 500 nM, 1 μM, 5 μM, 10 μM, 25 μM) for 24 h unless otherwise indicated.

### 4.2. Cell Viability Assay

HT-1080 or HEK293 cells (1 × 10^4^ cells/well) and HSF (5 × 10^3^ cells/well) were seeded in transparent 96-well plates in DMEM supplemented with 10% FBS. One day after plating, the cells were treated with DMSO (<0.1%, NT—nontreated cells) or increasing doses of MIPP or MOMIPP (1 × 10^−9^–25 × 10^−6^ M) for 24 h. On the day of the MTS experiment, the cells were examined under an inverted microscope before adding the reagent. Because the compounds at the highest concentration of 25 μM precipitated in the culture medium, we excluded them from the test. In the experiment with caspase inhibitor, the HT-1080 cells were incubated for 6 h and 24 h in the presence of MIPP or MOMIPP (1 μM) with or without Z-VAD(OMe)-FMK (10 μM) (no. sc-311561, Santa Cruz Biotechnology, Santa Cruz, CA, USA). After the indicated time, cell viability was examined using an MTS-based CellTiter96^®^ AQueous One Solution Cell Proliferation Assay (no. G3581, Promega, Madison, WI, USA) according to the manufacturer’s protocol. Briefly, 20 μL of MTS solution was pipetted into each well containing 100 μL of culture or culture medium (negative control) and incubated at 37 °C for 2 h (HT-1080) or 3 h (HEK293, HSF). Then, the absorbance was measured at 490 nm with the EnSpire microplate reader (PerkinElmer, Waltham, MA, USA). The IC_50_ values were calculated from semi-log plots of drug concentration against the viable cells using GraphPad Prism (v. 4.0.3). The plots were generated from the MTS results for serial dilutions of each compound, as indicated above.

### 4.3. Reactive Oxygen Species (ROS) Measurement

The ROS measurement was assayed by 2′,7′-Dichlorofluorescin diacetate (DCFH_2_-DA, no. D6883, Sigma Aldrich, St. Louis, MO, USA) [32]. The protocol was modified as described previously [33]. Briefly, HT-1080 cells (1 × 10^4^ cells/well) were cultured in a black-sided, transparent-bottom 96-well plate in DMEM without phenol red and supplemented with 1% FBS in the presence of (1) 0.1% DMSO (NT), (2) 1 μM MIPP or MOMIPP, (3) 30 μM oligomycin (no. 11342, Cayman Chemical, Ann Arbor, MI, USA), and (4) pretreated with 1 μM MIPP or MOMIPP for 1 h, followed by treatment with 30 μM oligomycin. The cells were treated with the indicated compounds for 4 h and 18 h. After incubation, the 25 μM nonfluorescent dye DCFH2-DA solution was freshly prepared in warm HBSS (no. 14025-059, Gibco^TM^, Thermo Scientific, Waltham, MA, USA) and added to the cells, which were then kept in the incubator for 45 min. Subsequently, the cells were washed twice with warmed HBSS to remove excess dye. Highly fluorescent dichlorofluorescein (DCF) is produced when ROS oxidizes this dye. The fluorescence was measured at Ex/Em = 485/535 nm using the EnSpire microplate reader (PerkinElmer, Waltham, MA, USA).

### 4.4. Analysis of Apoptosis

The activity of caspase-3 and -7 was used as a marker for cell apoptosis and was measured by the Apo-ONE^®^ Homogeneous Caspase-3/7 Assay (no. G7790, Promega, Madison, WI, USA) according to the manufacturer’s instructions. In the assay, caspase-3/7 cleaves a fluorescent DEVD peptide-rhodamine 110 substrate [(Z-DEVD)2-R110]. Briefly, the HT-1080 cells (4 × 10^3^ cells/well) were cultured in a 384-well plate with a clear bottom and black-sided in DMEM supplemented with 5% FBS. The arrangement of the experiment with caspase inhibitor Z-VAD(OMe)-FMK (10 μM) was the following: (1) cells treated with DMSO (<0.1%, NT), and (2) cells treated with 1 μM of MIPP or MOMIPP for 6 h and 24 h in the presence or absence of the pan-caspase inhibitor Z-VAD(OMe)-FMK (10 µM). The arrangement of the experiment with chloroquine (CQ, 60 μM, L10382, Invitrogen, Thermo Scientific, Carlsbad, CA, USA) was the following: (1) cells treated with DMSO (<0.1%, NT), (2) cells treated with 60 μM of CQ, and (3) cells treated with 1 μM of MIPP or MOMIPP for 15 h in the presence or absence of the CQ (60 µM). After treatment, 100 µL of Apo-ONE Homogeneous Caspase-3/7 reagent was added to each well containing 100 µL of cell culture medium and cells. The contents were gently mixed on a plate shaker for 30 min at room temperature. The fluorescence signal was recorded after 2 h at a 499 nm excitation wavelength and a 521 nm emission wavelength using the EnSpire fluorescence microplate reader (PerkinElmer, Waltham, MA, USA). The activity of the caspases was expressed as a percentage of the nontreated cells.

### 4.5. Mitochondrial Inner Membrane Potential (MMP) Measurement

The MMP was measured by a commercially available kit (Sigma Aldrich, MAK159, for live cells, St. Louis, MO, USA) that utilizes the cationic, lipophilic dye JC-10. It allows for the ratiometric analysis of the mitochondrial inner membrane potential, where a shift from orange (λ_EX_/λ_EM_: 540 nm/590 nm) to green fluorescence (λ_EX_/λ_EM_: 490 nm/525 nm) is indicative of compromised mitochondria. For this assay, the HT-1080 cells (1 × 10^4^ cells/well) were cultured in a black-sided, transparent-bottom 96-well plate in DMEM supplemented with 5% FBS in the presence of (1) DMSO (<0.1%, NT), (2) 1 μM staurosporine (STA, no. 328530010, Thermo Scientific, Waltham, MA, USA), and (3) 1 μM MIPP or MOMIPP for 6 h and 24 h. After incubation, 50 μL/well of the JC-10 dye loading solution was added to each well with cells. The plate was incubated for 45 min in the incubator. Then, 50 μL/well of Assay Buffer B was added to each well, and the fluorescence was monitored immediately. The results were calculated using the ratio of red to green fluorescence intensity.

### 4.6. Immunocytochemistry (IHC)

HT-1080 cells were cultured in an 8-well chamber slide (Nunc Lab-Tek II, Thermo Fisher Scientific, Denmark) at a density of 2 × 10^4^ cells per chamber. The following day, the cells were treated with (1) 0.1% DMSO, (2) 500 nM of rapamycin, (3) and 1 μL of MOMIPP for 24 h. The slides for immunocytochemical staining were prepared as described previously [33]. The antibodies used were as follows: first antibody: Atg8-LC3 (N-terminal) (overnight incubation in 4 °C, 1:1000, rabbit anti-human MAP1LC3A/B, AHP2167T, Bio-Rad, Hercules, CA, USA); and second antibody: donkey NorthernLights^TM^ anti-rabbit IgG-NL493 (2 h of incubation, 1:200, λ_EX_ = 493 nm, λ_EM_ = 514 nm, NL006, R&D Systems, Minneapolis, MN, USA). Finally, Hoechst (1 μg/mL, 10 min incubation, λ_EX_ = 352 nm, λ_EM_ = 454 nm) staining was performed. Before examination under the microscope, the preparations were coverslipped with an Aquatex mounting medium (Merck, Darmstadt, Germany). The pictures were taken by Leica TCS SP8 (Leica Microsystems, Heidelberg, Germany) with a HyVolution 2 high-resolution microscopy attachment. Leica LAS X microscope software (version 3.1.1.15751) was used to process the images.

### 4.7. Electron Microscopy

Cells were fixed with 2.5% glutaraldehyde overnight at 4 °C, washed in 0.1 M cacodylic buffer, and postfixed for 1 h in 1% OsO4. Next, the samples were dehydrated in a graded ethanol series for 10 min each (50%, 70%, 90%, 100%) and in propylene oxide, 2 × 10 min. Infiltration with resin was carried out by increasing the concentration of Poly/Bed 812 in 100% propylene oxide, and the mixture was left overnight at room temperature in a 1:1 ratio. The samples were transferred to pure resin, and polymerization was carried out at 60 °C for 3 days. Ultrathin sections of about 65 nm thick were cut and contrasted with uranyl acetate and lead citrate on the grids. The images were taken with a Jeol JEM 2100 HT TEM. For cutting, the Leica EM UC7 was used.

### 4.8. Statistical Analysis

Data are presented as the mean ± SEM of three independent experiments. Each treatment point was repeated (six times) in three independent technical replicates unless otherwise indicated. All statistical analyses were carried out using GraphPad Prism 7, with significance determined by one-way ANOVA followed by Sidak’s post hoc comparison tests, as detailed in the figure legends.

## Figures and Tables

**Table 1:** Values of IC50 for MIPP and MOMIPP on the growth of various cells.

Cells	MIPP IC_50_ [μM]	MOMIPP IC_50_ [μM]
HT1080	29.90	3.67
HEK293	1.29	16.28
HSF	0.56	9.70

## Data Availability

Dataset is available on request from the authors.

## Supplementary Materials

The following supporting information can be downloaded at https://www.mdpi.com/article/10.3390/ijms25116100/s1.
