# Supplementary material for: Studies on Autophagy and Apoptosis of Fibrosarcoma HT-1080 Cells Mediated by Chalcone with Indole Moiety
Source: Int J Mol Sci. 2024 Jun 1;25(11):6100. doi: 10.3390/ijms25116100 (PMC11172467; doi:10.3390/ijms25116100)

S1. The full purity analysis (TLC, HPLC) and confirmation of structures with spectral methods (UPLC-MS, ^{1}H NMR and ^{13}C NMR) of MIPP and MOMIPP.

MIPP

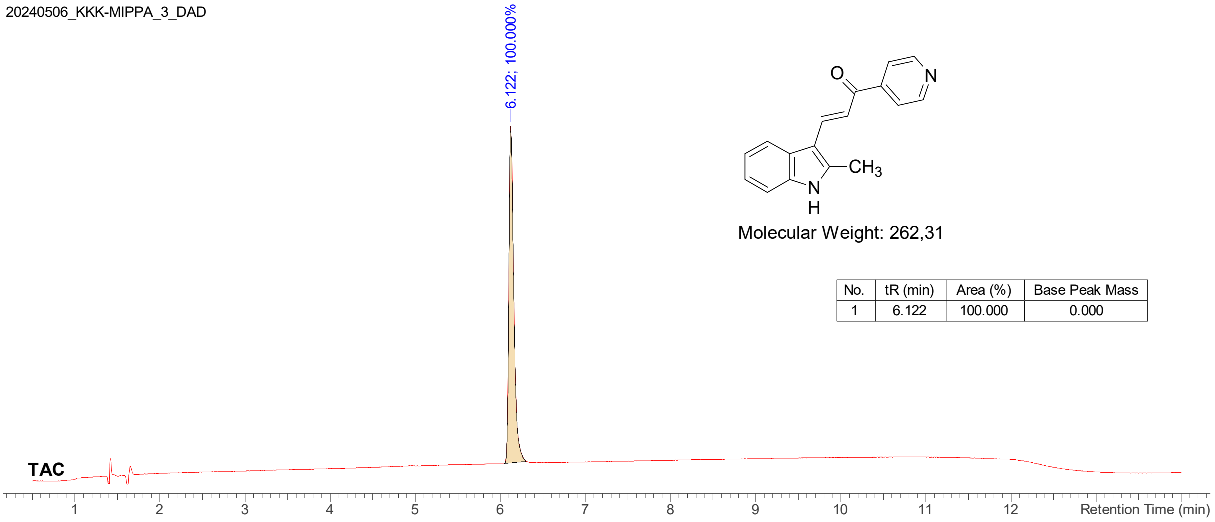

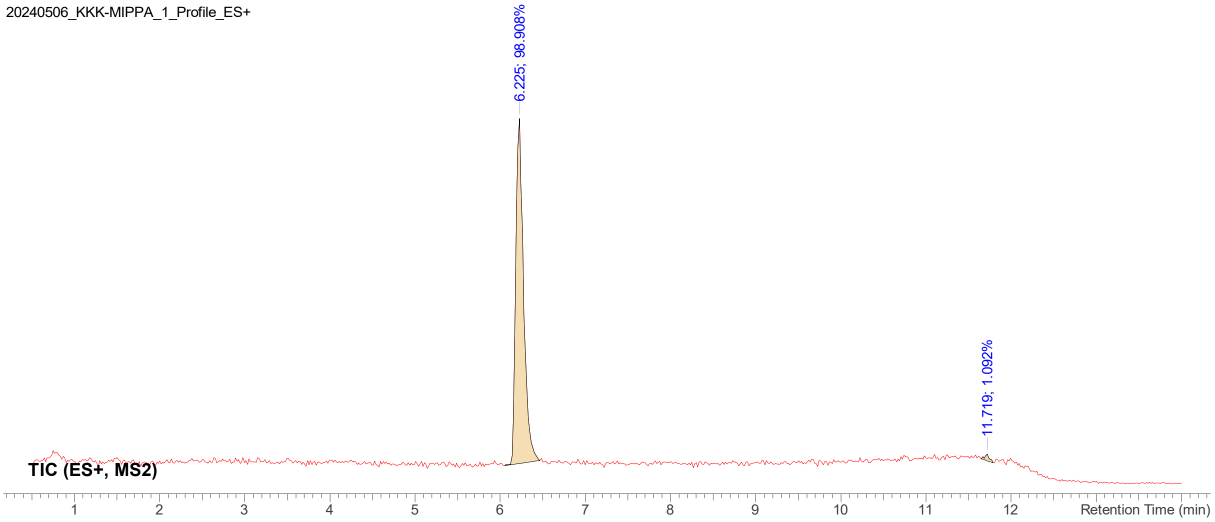

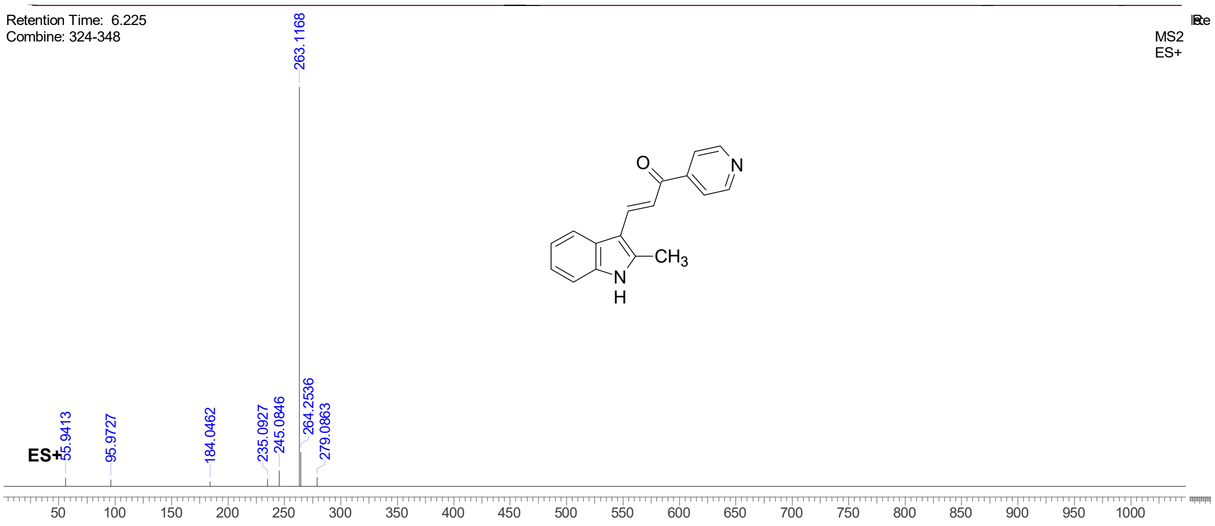

MIPP

Acquisition Time (sec)	1.9984	***Date***	07 May 2024 08:54:09			***Date Stamp***	07 May 2024 08:53:30		
File Name	C:\Users\Gosia\Downloads\MIPP_905_13_PROTON-1-1.jdf					***Frequency (MHz)***	500.16	***Nucleus***	1H
Number of Transients	8	***Origin***	ECA	***Original Points Count***	18757	***Owner***	delta	***Points Count***	32768
Pulse Sequence	single_pulse.jxp			***Solvent***	DMSO-d6	***Spectrum Offset (Hz)***	3251.0396	***Spectrum Type***	STANDARD
Sweep Width (Hz)	9385.89	***Temperature (degree C)***	20.000						

^{1}H NMR (500 MHz, DMSO-d_6) δ ppm 2.56 (s, 3 H) 7.14 - 7.21 (m, 2 H) 7.34 - 7.39 (m, 1 H) 7.44 (d, *J*=15.18 Hz, 1 H) 7.93 (d, *J*=5.73 Hz, 2 H) 8.03 (dd, *J*=5.87, 2.72 Hz, 1 H) 8.07 (d, *J*=15.18 Hz, 1 H) 8.77 (d, *J*=6.01 Hz, 2 H) 11.67 - 12.29 (m, 1 H)

MIPP_905_13_PROTON-1-1.jdf

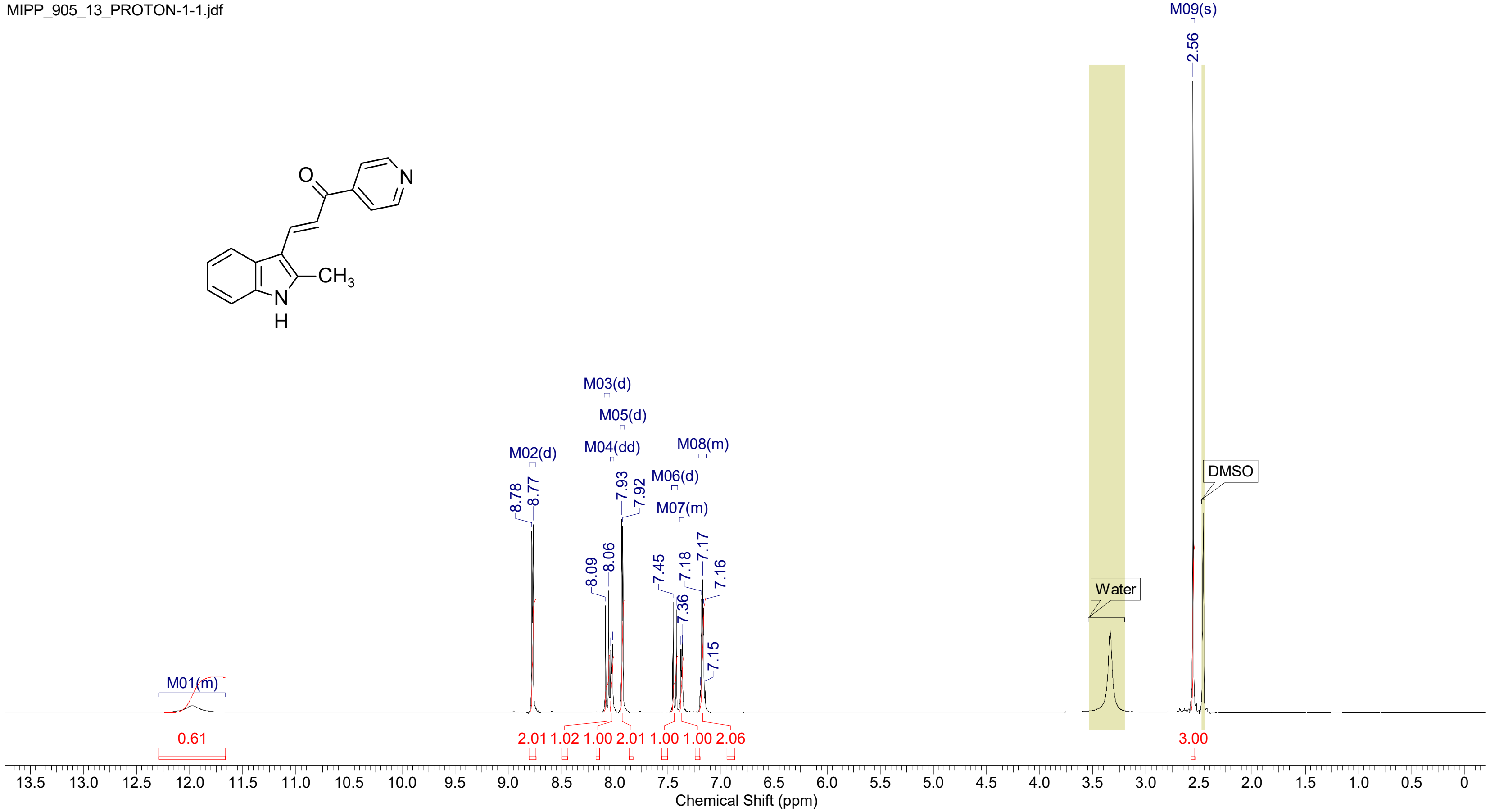

MIPP_905_13_PROTON-1-1.jdf

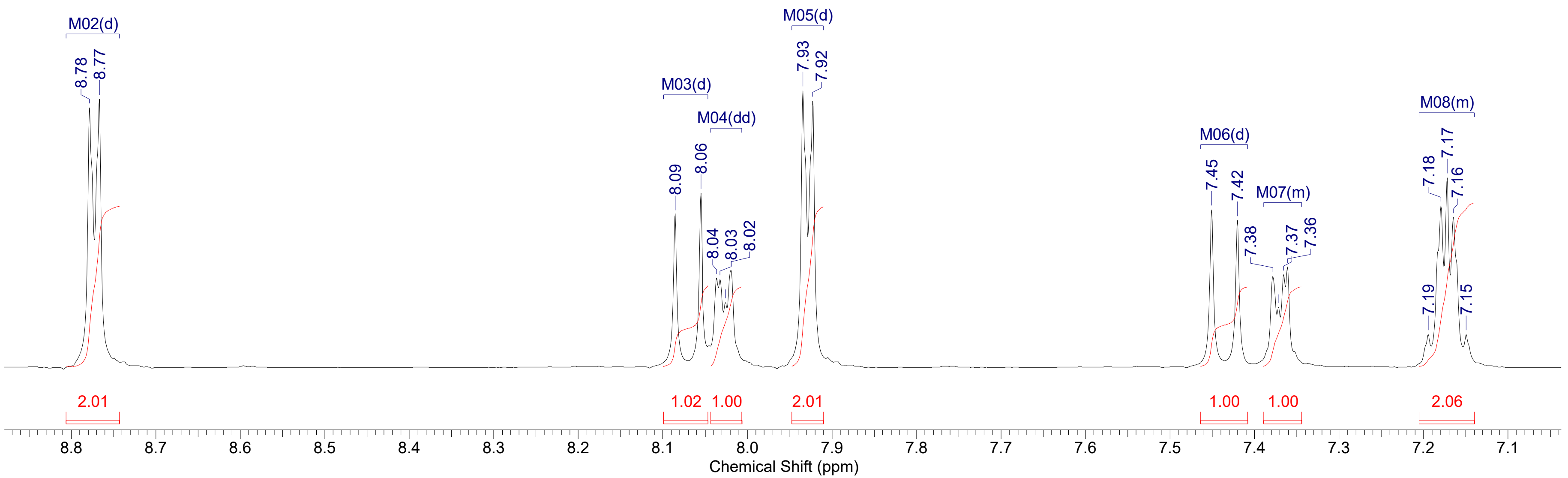

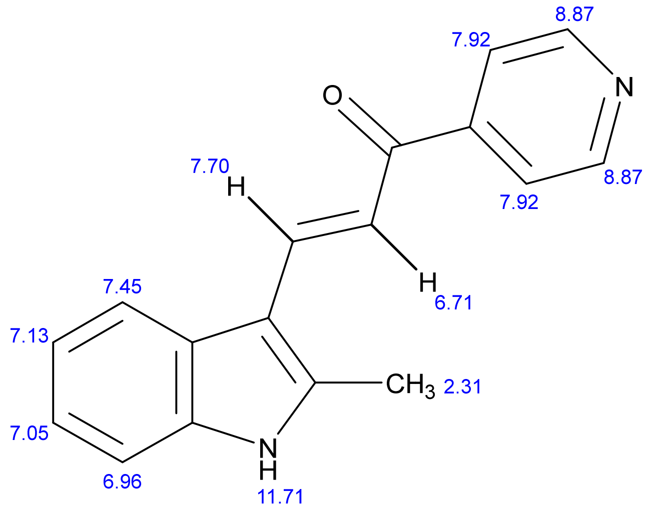

Estimation quality is indicated by color: good, medium, rough

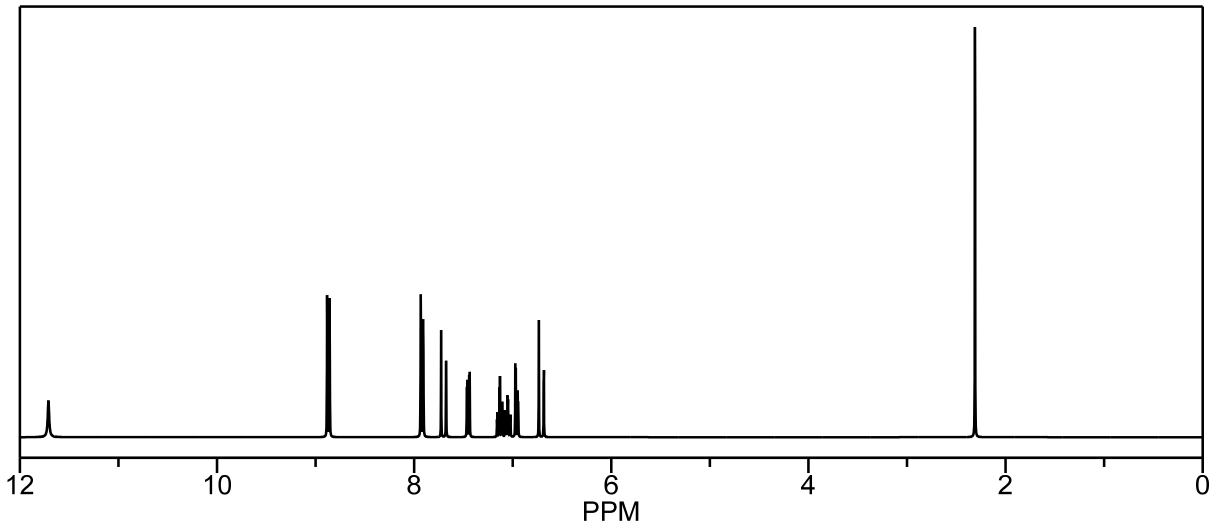

Acquisition Time (sec)	1.9996	***Date***	07 May 2024 09:46:42			***Date Stamp***	07 May 2024 08:54:39
File Name	C:\Users\Gosia\OneDrive\Pulpit\MIPP-widma\MIPP_905_13_CARBON-1-1.jdf					***Frequency (MHz)***	125.77
Nucleus	13C	***Number of Transients***	1024	***Origin***	ECA	***Original Points Count***	78604
Owner	delta	***Points Count***	131072	***Pulse Sequence***	carbon.jxp	***Solvent***	DMSO-d6
Spectrum Offset (Hz)	12576.5293	***Sweep Width (Hz)***	39310.18	***Temperature (degree C)***	20.000		

MIPP_905_13_CARBON-1-1.jdf

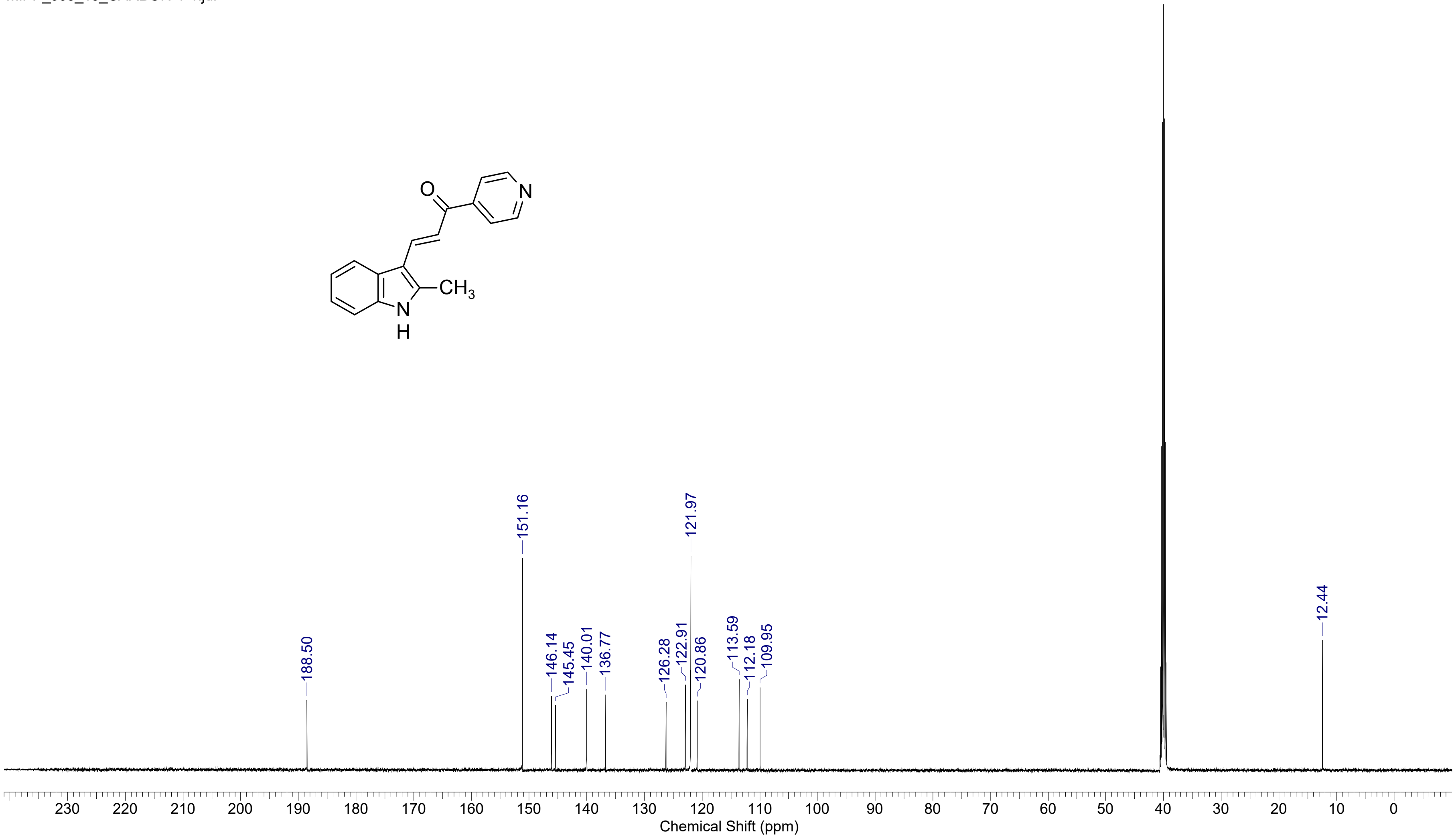

MOMIPP

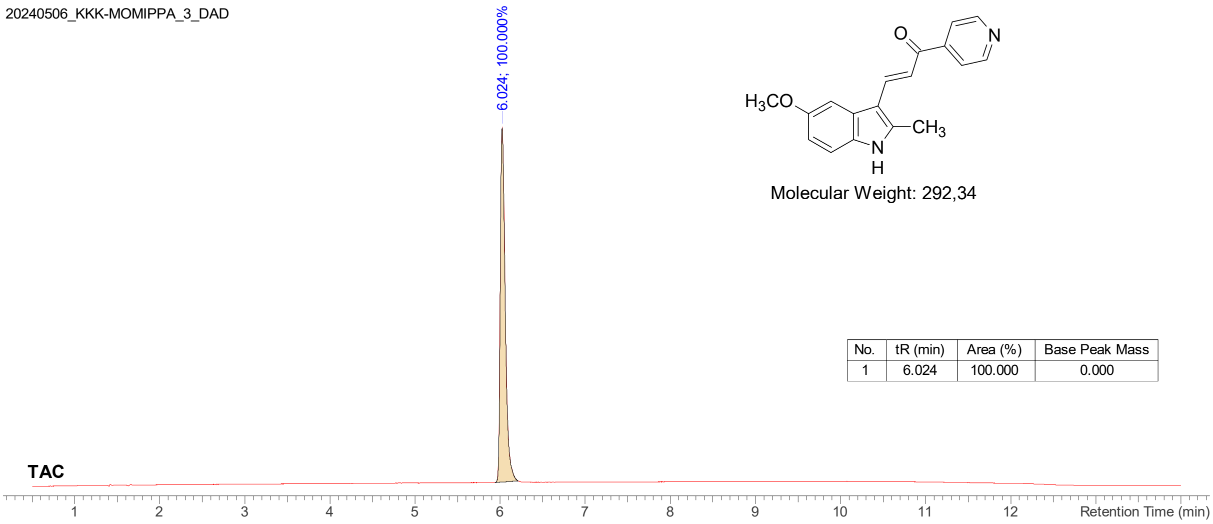

No.	tR (min)	Area (%)	Base Peak Mass
1	6.024	100.000	0.000

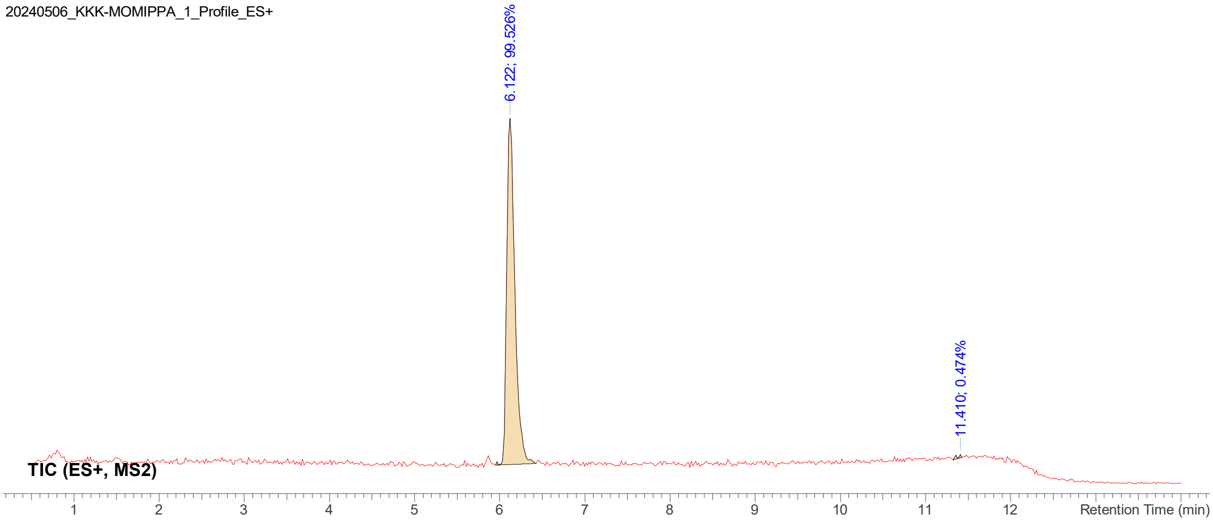

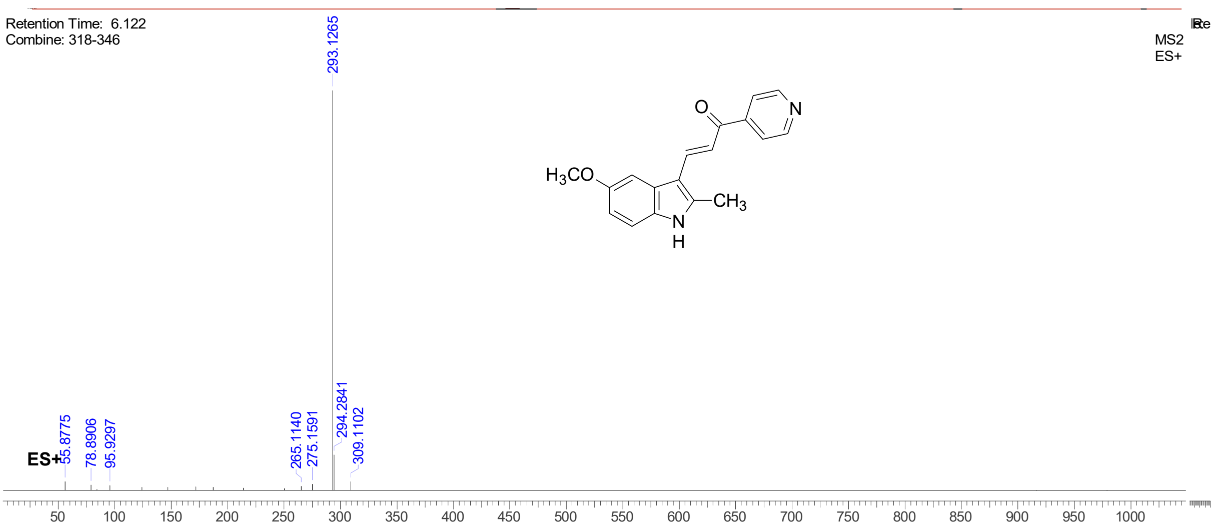

MOMIPP

Acquisition Time (sec)	1.9984	Date	07 May 2024 09:50:15			Date Stamp	07 May 2024 09:49:37
File Name	C:\Users\Gosia\OneDrive\Pulpit\MIPP-widma\MOM1PP_905_13_PROTON-1-1.jdf					Frequency (MHz)	500.16
Nucleus	1H	Number of Transients	8	Origin	ECA	Original Points Count	18757
Owner	delta	Points Count	32768	Pulse Sequence	single_pulse.jxp	Solvent	DMSO-d6
Spectrum Offset (Hz)	3251.0396	Spectrum Type	STANDARD	Sweep Width (Hz)	9385.89	Temperature (degree C)	20.000

^{1}H NMR (500 MHz, DMSO-d_6) δ ppm 2.53 (s, 3 H) 3.82 (s, 3 H) 6.80 (dd, J=8.74, 2.15 Hz, 1 H) 7.27 (d, J=8.59 Hz, 1 H) 7.32 (d, J=15.47 Hz, 1 H) 7.39 (d, J=2.29 Hz, 1 H) 7.90 (d, J=5.73 Hz, 2 H) 8.05 (d, J=15.18 Hz, 1 H) 8.77 (d, J=6.02 Hz, 2 H) 11.45 - 12.16 (m, 1 H)

MOM1PP_905_13_PROTON-1-1.jdf

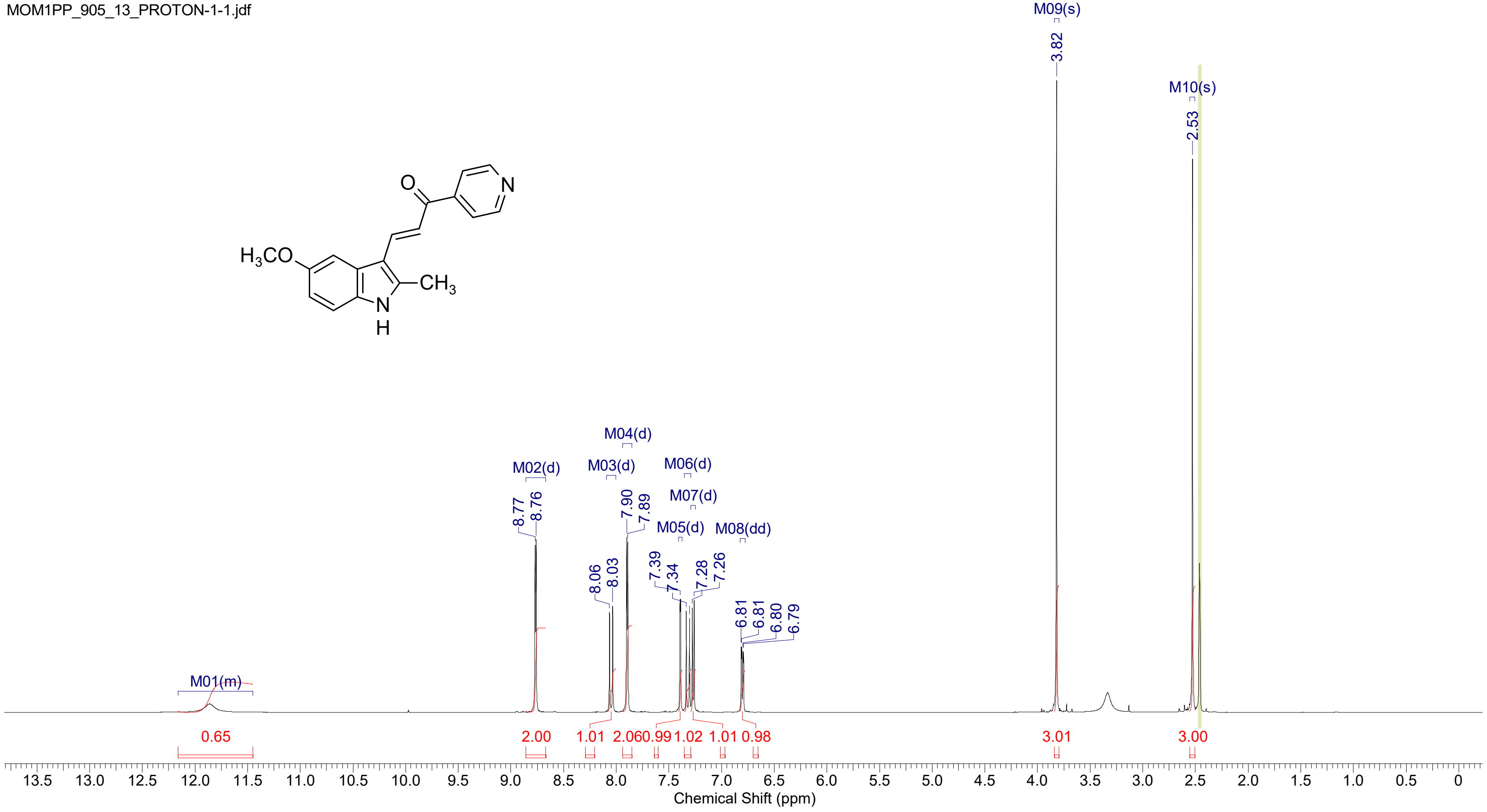

MOM1PP_905_13_PROTON-1-1.jdf

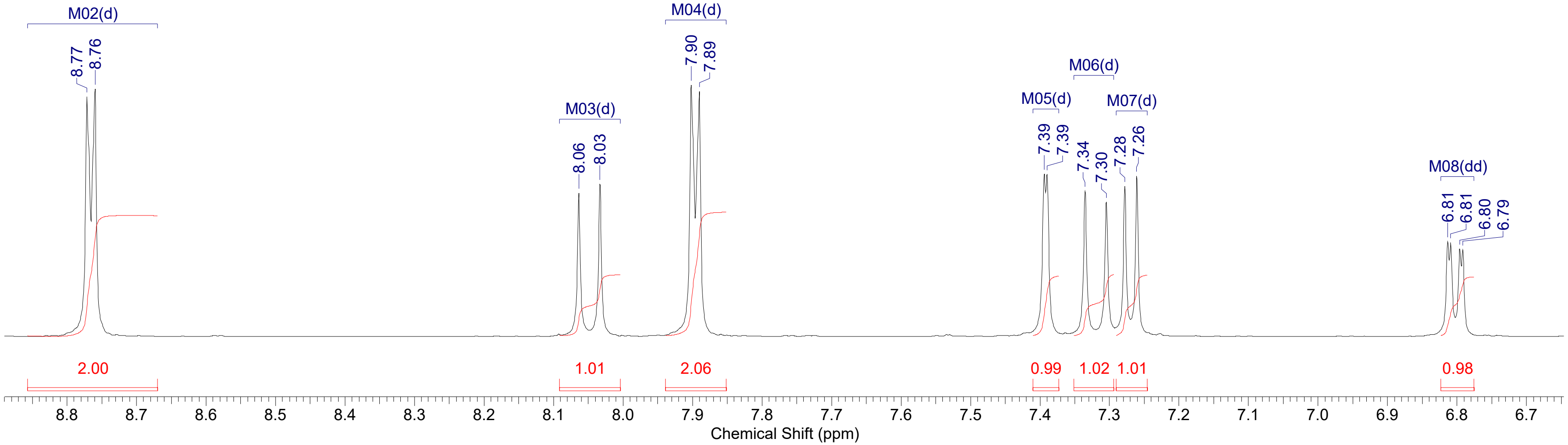

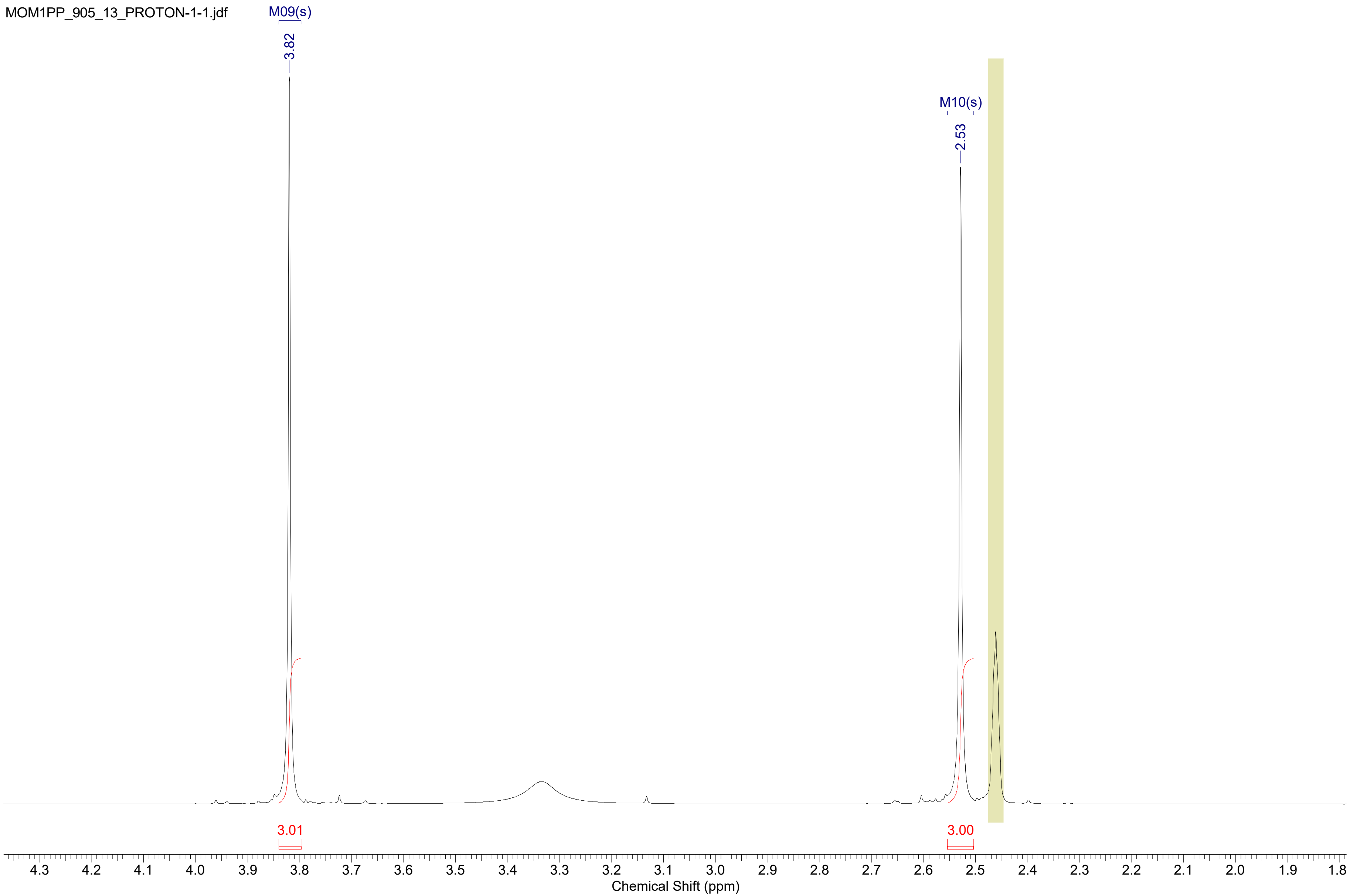
MOM1PP_905_13_PROTON-1-1.jdf
M09(s)
3.82
M10(s)
2.53
3.01
3.00
4.3 4.2 4.1 4.0 3.9 3.8 3.7 3.6 3.5 3.4 3.3 3.2 3.1 3.0 2.9 2.8 2.7 2.6 2.5 2.4 2.3 2.2 2.1 2.0 1.9 1.8
Chemical Shift (ppm)

Acquisition Time (sec)	1.9996	***Date***	07 May 2024 10:42:49			***Date Stamp***	07 May 2024 09:50:46
File Name	C:\Users\Gosia\OneDrive\Pulpit\MIPP-widma\MOM1PP_905_13_CARBON-1-1.jdf					***Frequency (MHz)***	125.77
Nucleus	13C	***Number of Transients***	1024	***Origin***	ECA	***Original Points Count***	78604
Owner	delta	***Points Count***	131072	***Pulse Sequence***	carbon.jxp	***Solvent***	DMSO-d6
Spectrum Offset (Hz)	12576.5293	***Sweep Width (Hz)***	39310.18	***Temperature (degree C)***	20.200		

MOM1PP_905_13_CARBON-1-1.jdf

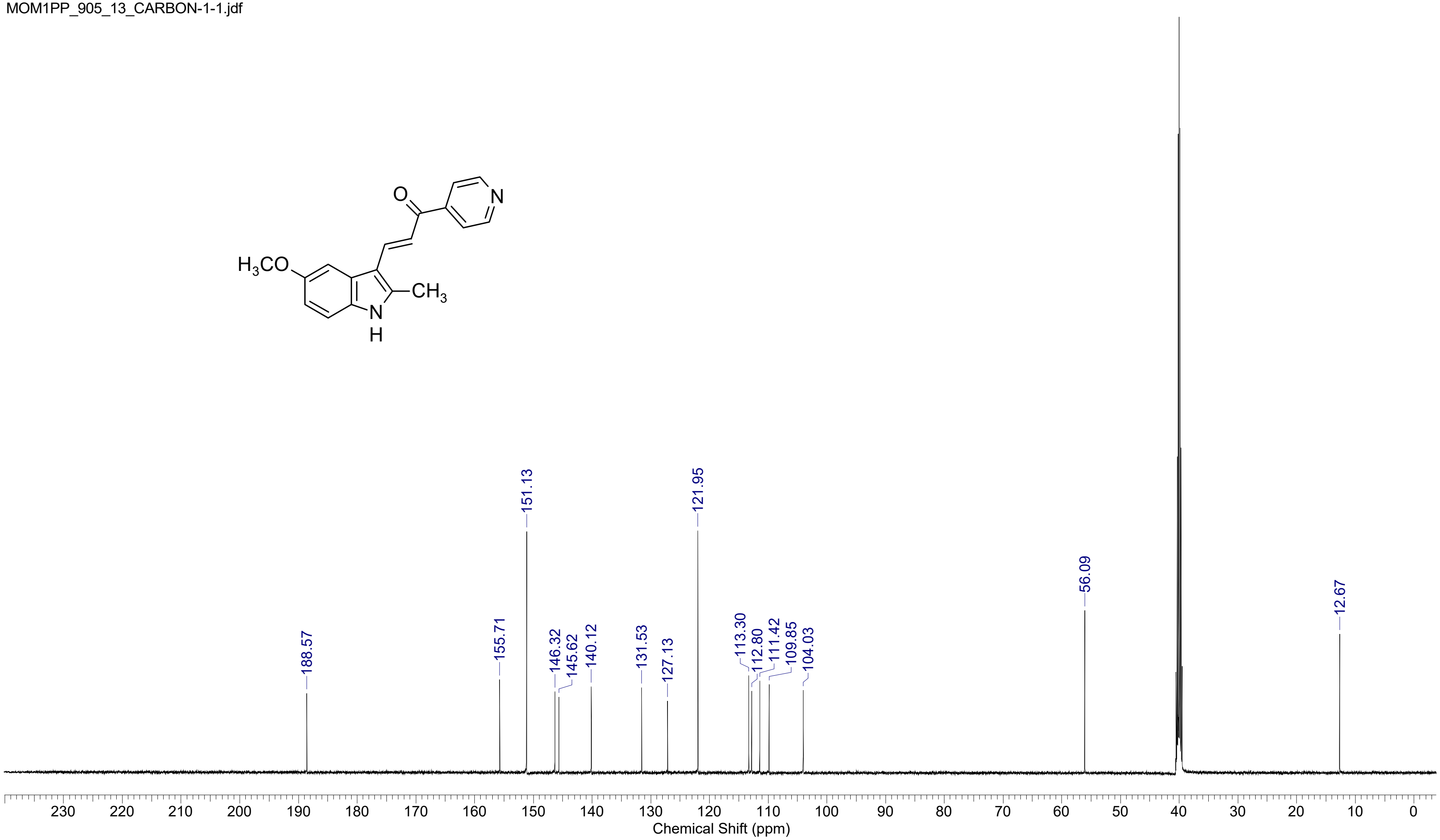

Supplement: Supplementary file 1 [file ijms-25-06100-s001.zip › ijms-2988700-supplementary.pdf]
